# Keeping the Faith: African American Faith Leaders’ Perspectives and Recommendations for Reducing Racial Disparities in HIV/AIDS Infection

**DOI:** 10.1371/journal.pone.0036172

**Published:** 2012-05-16

**Authors:** Amy Nunn, Alexandra Cornwall, Nora Chute, Julia Sanders, Gladys Thomas, George James, Michelle Lally, Stacey Trooskin, Timothy Flanigan

**Affiliations:** 1 Warren Alpert Medical School of Brown University, Division of Infectious Diseases, Providence, Rhode Island, United States of America; 2 The Miriam Hospital, Division of Infectious Diseases, Providence, Rhode Island, United States of America; 3 University of Pennsylvania, Center for AIDS Research Community Advisory Board, Philadelphia, Pennsylvania, United States of America; 4 Council for Relationships, Philadelphia, Pennsylvania, United States of America; 5 University of Pennsylvania, Division of Infectious Diseases, Philadelphia, Pennsylvania, United States of America; Vanderbilt University, United States of America

## Abstract

In Philadelphia, 66% of new HIV infections are among African Americans and 2% of African Americans are living with HIV. The city of Philadelphia has among the largest numbers of faith institutions of any city in the country. Although faith-based institutions play an important role in the African American community, their response to the AIDS epidemic has historically been lacking. We convened 38 of Philadelphia’s most influential African American faith leaders for in-depth interviews and focus groups examining the role of faith-based institutions in HIV prevention. Participants were asked to comment on barriers to engaging faith-based leaders in HIV prevention and were asked to provide normative recommendations for how African American faith institutions can enhance HIV/AIDS prevention and reduce racial disparities in HIV infection. Many faith leaders cited lack of knowledge about Philadelphia’s racial disparities in HIV infection as a common reason for not previously engaging in HIV programs; others noted their congregations’ existing HIV prevention and outreach programs and shared lessons learned. Barriers to engaging the faith community in HIV prevention included: concerns about tacitly endorsing extramarital sex by promoting condom use, lack of educational information appropriate for a faith-based audience, and fear of losing congregants and revenue as a result of discussing human sexuality and HIV/AIDS from the pulpit. However, many leaders expressed a moral imperative to respond to the AIDS epidemic, and believed clergy should play a greater role in HIV prevention. Many participants noted that controversy surrounding homosexuality has historically divided the faith community and prohibited an appropriate response to the epidemic; many expressed interest in balancing traditional theology with practical public health approaches to HIV prevention. Leaders suggested the faith community should: promote HIV testing, including during or after worship services and in clinical settings; integrate HIV/AIDS topics into health messaging and sermons; couch HIV/AIDS in social justice, human rights and public health language rather than in sexual risk behavior terms; embrace diverse approaches to HIV prevention in their houses of worship; conduct community outreach and host educational sessions for youth; and collaborate on a citywide, interfaith HIV testing and prevention campaign to combat stigma and raise awareness about the African American epidemic. Many African American faith-based leaders are poised to address racial disparities in HIV infection. HIV prevention campaigns should integrate leaders’ recommendations for tailoring HIV prevention for a faith-based audience.

## Introduction

African Americans account for 13% of the US population [1], but over 50% of new HIV/AIDS diagnoses [2]. Infection rates for African Americans are seven times those of Whites [2]. Philadelphia’s racial disparities in HIV infection are especially marked; nearly 70% of new HIV infections are among African Americans and 2% of Philadelphia’s African American population is living with HIV/AIDS [3].

Risk behaviors such as condom use, drug use and number of lifetime sexual partners do fully not explain racial disparities in HIV infection [4], and a growing body of evidence suggests that social and structural factors such as poverty, stigma, incarceration and sexual networks contribute to racial disparities in HIV infection [5]. Behavioral interventions have failed to stem the HIV/AIDS epidemic among African Americans in the US and do not address many of the aforementioned social and structural factors that contribute to HIV infection among African Americans [5].

Research finds that stigma associated with HIV/AIDS [6] and homophobia [7] is more prevalent among African Americans than individuals of other races. Stigma has hindered efforts to reduce racial disparities in HIV infection among African Americans and has been associated with HIV risk behaviors [8]–[11] and barriers to HIV testing among African Americans [8], [9], [12]–[15].

Religious institutions are often cited as the cornerstone of the African American community; African American churches have a more than 200-year history of providing social and support services and played critical roles in the US civil rights movement [16]. Churches in particular continue to have important roles in African American community mobilization and serve as important community and political meeting centers [17], [18]. A recent nationwide survey [19] finds African Americans are the most religiously committed racial or ethnic group in the nation: nearly 80% of African Americans report that religion plays important roles in their lives, compared to 56% of all US adults. More than half attend religious services more than once a week, 76% pray on a daily basis, and 88% indicate they are certain God exists [19]. African Americans are far more likely than other religious groups to participate in some sort of organized religious service and express a much higher degree of comfort with religious institutions’ engagement in political and public life [19]. Other research underscores the importance of spirituality for African Americans living with HIV/AIDS [20]. While these phenomena suggest that African American faith-based institutions are uniquely poised to address the HIV/AIDS epidemic, the response to the AIDS epidemic by African American faith institutions has been lacking. This has been attributed to several factors, including the fact that HIV has been associated with homosexuality [21]–[26] and immoral behavior [27], [28] within the African American faith community.

Although the Centers for Disease Control and Prevention (CDC) [29] and President Obama’s National AIDS Strategy [30] have called for new partnerships with faith-based institutions, little is known about how to effectively engage African American faith leaders in HIV prevention. Two recent reviews of congregation-based programs to address HIV/AIDS highlight the paucity of information on this topic [31], [32], the importance of a community-based participatory research (CBPR) approach for developing HIV/AIDS programs, and the historical role of stigma in hindering faith-based institutions’ response to the epidemic [32]. The few examples of faith-based HIV interventions developed with African American churches attribute positive results to community-based, culturally relevant approaches [33]–[42] and two other articles highlight the increasing willingness of faith-based institutions to engage in HIV prevention [43], [44]. A few African American churches have had HIV/AIDS programs since the late 1980s [34] and *The Balm in Gilead*, a non-profit organization, has been conducting HIV/AIDS programs in faith-based institutions for many years [45].

In spite of the aforementioned challenges and opportunities for engaging African American faith institutions in HIV prevention, little research examines the perspectives of Pastors and Imams about barriers to addressing HIV/AIDS and their recommendations for how to enhance HIV prevention in faith-based settings.

Philadelphia, PA has a large African American population, some of the widest racial disparities in HIV infection in the country, HIV infection rates five times the national average [46] and is home to many of the country’s first African American churches, many of which are still operational today.

We conducted in-depth interviews and focus groups with prominent Philadelphia African American religious leaders about their knowledge about HIV transmission, Philadelphia’s HIV/AIDS epidemic, and their opinions about the social, behavioral and structural drivers of HIV infection. To our knowledge, no body of research has specifically solicited the normative recommendations of faith leaders about how they can contribute to HIV prevention and reduce racial disparities in HIV infection; we therefore also solicited normative recommendations for how to enhance HIV prevention in a faith-based context.

## Methods

### Ethics Statement

This study protocol was approved by the Miriam Hospital Institutional Review Board. Because risks associated with this study were minimal, the Miriam Hospital IRB recommended we obtain verbal informed consent. Prior to interviews and focus group commencement, participants were informed of the details of the study and provided an opportunity to decline to participate. Moderators wrote down the names of all participants providing verbal informed consent prior to focus group commencement.

### Recruitment and Enrollment

Philadelphia Mayor Nutter’s Office of Faith-based Initiatives and investigators affiliated with Brown University convened key informant interviews and five focus groups among African American Pastors and Imams in May and June 2010. Participants were recruited based on their leadership roles in predominantly African American houses of worship across the city of Philadelphia. Focus group participants convened for a breakfast meeting, provided verbal informed consent, and received a $100 gift card for participating.

We used purposeful sampling, which selects the study sample based on respondents’ unique characteristics. This strategy is particularly useful for gaining insights of key opinion leaders or stakeholders [47], [48]. We invited many Pastors and Imams from Philadelphia’s largest faith institutions and others known for their social outreach programs to participant in key informant interviews and focus groups. Key informant interviews informed our approach to the project and larger focus group discussions. To ensure as much diversity as possible in focus group composition, each focus group was comprised of individuals from diverse Christian and Muslim denominations.

### Focus Groups

We used a grounded theory qualitative interviewing approach in which data informs development of theory and subsequent data analysis [49], [50]. We used a semi-structured interview guide to conduct focus group discussions. The focus group interview guide was informed by key informant interviews among over twenty local African American faith leaders, peer-reviewed literature related to the local and national HIVAIDS epidemics [2], [15], [46], [51]–[53], engaging faith leaders in HIV prevention [39], [52], other literature highlighting the importance of spirituality and churches in African American culture [19], [20], [54]; and the opinions and experience of the Director of the Mayor’s Office of Faith Based Initiatives. The focus group guide included questions about faith leaders’ knowledge of HIV transmission and the local Philadelphia epidemic, factors contributing to Philadelphia’s HIV/AIDS epidemic, existing HIV/AIDS programs in their congregations, challenges and opportunities for addressing HIV/AIDS in a faith-based context, and leaders’ normative suggestions for how the faith community can enhance HIV prevention in Philadelphia. The semi-structured focus group guide consisted of open-ended questions, was designed to elicit a wide range of responses, and allowed the respondent and moderator to freely introduce new topics as the conversation flowed [55]. Trained African American moderators conducted focus groups.

### Analysis

Focus groups discussions lasted approximately an hour and a half, were digitally recorded, captured electronically on computer software, and professionally transcribed. Transcripts were checked for accuracy, and identifying information was removed from transcripts. In accordance with grounded theory, data collection and theory building were iterative; data collection informed development of theories and those theories informed subsequent data analysis [49]. The first reading of transcripts was deductive and allowed coders to read and capture participants’ answers to individual questions. The second review looked for recurring themes in the text and informed development of a coding scheme used to analyze transcript data [56]. This coding scheme was discussed in detail among data analysts and the Principal Investigator. In accordance with grounded theory, as new understandings about barriers and opportunities for addressing racial disparities in HIV infection with faith leaders emerged, and as new themes emerged, these issues were integrated into the theory building process, and the coding and final analytical scheme. Transcripts were then coded by three analysts to enhance validity and concordance; discrepancies in coding were discussed and resolved among analysts. Upon concluding this iterative analytical process, primary findings were synthesized and summarized in analytic memos. To enhance the reliability and validity of our findings, we also conducted “member checks,” a process in which we shared our interpretations of primary findings with several focus group participants. These member checks helped us sharpen our understanding and presentation of the following results, and normative recommendations in particular.

## Results

Thirty-eight faith leaders participated in the focus groups. Table 1 provides demographic and religious affiliations of the participants. Emergent themes were grouped into two categories: 1) barriers and challenges to engaging the African American faith community in HIV/AIDS prevention programs (Table 2); and 2) opportunities and recommendations from faith leaders for engaging the faith community in HIV prevention (Table 3). We elaborate on those themes here.

**Table 1:** Demographic Information and Religious Affiliation of Focus Group Participants.

Variable	N = 38	Percentage
Gender		
Male	27	71
Female	11	29
Denomination		
Baptist	15	39
African Methodist Episcopalian (AME)	6	16
Muslim	5	13
Non-denominational	5	13
Methodist	2	6
Pentecostal	3	6
Evangelical	1	3
Jewish	1	3

**Table 2:** Barriers and Challenges to Engaging African American Faith-Based Institutions in HIV Prevention.

Theme	Example of Barrier or Challenge
Faith leaders understand how HIV is transmitted but were unaware ofgravity of local HIV/AIDS epidemic	• Faith leaders were unaware of racial disparities and gravity of local epidemic• Faith leaders were unaware of city’s micro-epidemics and high incidence in their institutions’ neighborhoods
Discussing human sexuality in faith settings presents challenges	• Leaders perceived that discussing human sexuality may be inappropriate in faith settings• Leaders believed that many congregants may believe discussing human sexuality is inappropriate in faith settings
Homophobia and stigma inhibit discussion of HIV/AIDS among AfricanAmerican faith leaders	• Fear of being perceived as gay prevents male faith leaders from discussing HIV/AIDS• Leaders believe their congregants believe that HIV/AIDS is a gay disease• Leaders report that African Americans sometimes prefer not to discuss difficult topics, including HIV/AIDS
Leaders perceive that addressing reproductive health issues, includingHIV/AIDS, may conflict with theological traditions	• Leaders report apprehension about implementing sexual education programs in faith settings• Leaders believe HIV prevention messages encouraging condom use conflict with institutional commitments to “abstinence only”
Addressing HIV/AIDS presents financial and resource challenges forfaith-based institutions	• Leaders perceive that discussing HIV may negatively influence tithing and impact their institutions’ economic welfare• HIV/AIDS prevention programs require financial investments some institutions do not have• Long-term commitments to HIV prevention require sustained programmatic commitments
Pastoral experience, reputation, and age impact clergy’s willingness toaddress HIV/AIDS	• Young or less experienced clergy members feel less confident about discussing controversial topics such as HIV/AIDS

**Table 3:** Faith Leaders’ Recommendations for Enhancing HIV/AIDS Prevention in Faith-Based Institutions.

Theme	Specific Recommendations
Enhance leadership and advocacy efforts of African AmericanClergy related to HIV prevention	• Educate faith leaders about local epidemic to promote more widespread engagement in HIV prevention• Lead by Example: Faith leaders should undergo HIV testing to destigmatize and encourage widespread testing• Leaders should openly discuss HIV/AIDS with congregants
Normalize HIV testing and conversations about human sexualityto reduce HIV/AIDS stigma	• Promote and normalize routine HIV testing• Divorce HIV testing discussions from conversations about sexual orientation and sin• Support candid institutional and community dialogue about sexual and reproductive health• Frame HIV as a public health and human rights issue rather than a sexual orientation issue• Couch conversations about HIV/AIDS in the context of human healing rather than sexuality
HIV/AIDS Prevention in faith institutions requires diverseapproaches and must be tailored to individual institutions	• Host HIV testing events• Preach about HIV/AIDS and HIV testing from the pulpit• Utilize scripture to teach about HIV/AIDS and to reduce stigma• Host HIV/AIDS discussions in small non-Sunday forums• Incorporate abstinence into HIV prevention messages• Create HIV/AIDS ministries• Host community HIV testing and education events• Utilize social media outlets to reach youth with HIV/AIDS prevention and education messages• Engage the media in HIV prevention and awareness
Interfaith Collaboration will Enhance the Local HIV/AIDSPrevention Efforts	• Faith leaders can, should and are willing to work together on HIV prevention campaigns• Convene regular meetings of diverse faith leaders• Convene a city-wide HIV/AIDS prevention program with clergy

### Barriers and Challenges to Engaging the African American Faith Community in HIV/AIDS Education and Prevention Programs

#### Knowledge about HIV/AIDS and the local epidemic

Participants generally understood how HIV is transmitted, but were surprised to learn about the gravity of Philadelphia’s local epidemic. One Imam’s remark highlighted this common theme:

I’m amazed how little I know about [HIV in Philadelphia]. I was informed today with new information. It’s not as though I’m not listening to the news, but I would not have identified AIDS to be any more of a problem than cancer or some of the other diseases people are dying from. I did not know it was on this level.

#### Discussing human sexuality in faith contexts

Many participants concurred that discussing human sexuality in religious contexts was the greatest barrier to engaging faith communities in HIV prevention efforts. Several respondents reflected:

I find that talking about sexuality at church is a very tricky thing, not even just with the homosexuality but heterosexual sexuality.

In the Islamic faith, the barrier is sticking to the deen [authority of and submission to Allah], that’s where all emphasis is. It doesn’t even really address AIDS because you’re not supposed to have sex. Of course you’re going to have sex, but we just haven’t found ways to address it, or it just hasn’t been discussed. If it’s discussed, it’s within small groups where men discuss it among themselves. But they’re not coming up with a solution to get everybody involved and bring it to the forefront of any discussion.

It’s difficult to talk about HIV at church because we have defined what we will accept as the proper language, the proper subject, and the proper issues to talk about. Sex and HIV are subjects that make many uncomfortable.

#### Homophobia

Participants also commented extensively that homophobia and fear of being perceived as gay prevents many African American clergy from discussing HIV/AIDS:

People are afraid they’ll be thought of as gay. I think it’s the biggest thing with African American men. If AIDS weren’t a disease that first attacked the gay community, African American men would probably have less of a problem with it. But African American men do not want anybody to think that they are gay.

Let me talk about stigma for a moment. The big elephant in the room that created major problems and stigma for religious groups across the board is the belief that HIV/AIDS is a gay disease. That creates the fear that any man who comes forth will be labeled as gay, whether he has a family or not. Being gay is looked down on and frowned upon. There are a lot of other myths mixed in for women, such as being perceived as sexually promiscuous. The sexual aspect of this disease is big for the theological and biblical community.

On the other hand, a few respondents believed HIV/AIDS stigma was no longer a major barrier to addressing HIV/AIDS in the faith community. One participant commented:

I don’t find that there’s much stigma anymore. I think the stigma is not as pronounced on the street as much as we think. I even find the stigma has decreased in the church because many churches lost musicians and choir directors to AIDS.

#### Balancing sexual education with theology

Pastors and Imams commonly cited challenges with balancing theological messages with candid conversations about human sexuality and reproductive health:

One time my pastor spoke to young people about sex, mentioning using protection. I was sitting in the clergy row; you could feel the heat! I was surprised he said that. Comments from the clergy highlighted they were opposed to that. It’s a tightrope walk.

There’s a very delicate line of seemingly being open and receptive to hearing what’s on someone’s mind and being perceived as encouraging sexual activity.

Many respondents commented that discussing condoms and sexual education in faith settings conflicted (or could be perceived as conflicting by faith institutions’ elders) with church or mosque commitments to promoting abstinence until marriage:

In the faith community, we’ve taken positions promoting abstinence for so long that we don’t want to mention condoms because people may think we’re saying “You should be having promiscuous sex.”

I think it’s a very real issue, one that at some point the clergy has to deal with: the reality that people are having sex whether you tell them to abstain or not. I’ve had this debate over and over again with our youth leadership. Half of them want to tell kids to put a condom on, to protect themselves. But some of them say “If you’re telling them to protect themselves, then you telling them it’s okay to have sex.”

#### Silence about HIV/AIDS

Numerous respondents commented that a major barrier to addressing the HIV/AIDS epidemic was a tacit “code of silence” about HIV/AIDS in the African American community.

There is a code of silence in the Black community, which is our downfall with HIV.

Generally speaking, churches, pastors, religious leaders, have been silent. Or they’ve done what the bishop’s said, they’ve taken the position that AIDS is God’s curse on homosexual people. It’s ludicrous to think that. We can no longer be silent. The majority of our religious communities never raised the issue, either in preaching, or teaching, or in-group sessions. It’s very minimal.

As black houses of worship, churches, mosques, etc., we’ve had that credibility in some areas and the trust level in some areas. But in this area, perhaps because of our silence, we’ve not gained trust and credibility.

Another Pastor elaborated on the cultural roots of silence about HIV/AIDS:

The African American community has a cultural disposition not to talk about things that are painful and difficult. We don’t talk about people who are drug users in our families or infidelity in marital relationships. We don’t talk about sexuality, period. We try to be happier. We’ve got enough stress. Let’s talk about nice things. What’s driving this epidemic is our inability to talk about difficult topics, much less about HIV/AIDS.

One Imam commented that the Islamic community has not embraced HIV/AIDS as an important domestic social priority.

When I was looking at how the Islamic community has responded to AIDS, I pulled up a lot of different reports. Each dealt with what Muslims were doing in other countries; very little was being done in America. That’s the failure of the Islamic community: to even want to acknowledge that we have an AIDS problem.

#### Resource challenges and sustainability of HIV/AIDS programs

Participants identified several resource challenges associated with addressing HIV/AIDS in faith contexts. First, there was a common perception that discussing HIV/AIDS from the pulpit could have detrimental financial implications for faith institutions. One Pastor explained that discussing controversial topics such as sexuality and HIV/AIDS might affect church attendance and tithing:

If you talk about HIV, congregants may say “That ain’t got nothing to do with me.” That’s not actually going to inspire people, to come to church or to give their tithes and offers. It just won’t! That connection is not there.

Secondly, many participants explained that they need more financial resources to develop and sustain HIV/AIDS programs. Two pastors explained:

One of the things preventing people from getting involved is not so much attitudes, but just time and resources.

The problem of HIV/AIDS cannot be solved unless there’s money. Money is the acid test. Churches cannot do it alone, but the churches have to lead the fight because this is a civil rights issue. Churches need the local, state and federal governments’ monies. We’re talking about minorities being disproportionately infected and afflicted by this problem. The only way it can be solved, is to have money, or else the rich will live and the poor will die. That’s what’s happening. We can’t begin to talk about solving the problem until we find the money.

While many faith leaders identified budget and resource constraints as barriers to hosting regular HIV prevention events, many agreed on the importance of sustained engagement from the faith community:

HIV prevention should be an ongoing thing if we want to make an impact in our neighborhoods and our churches.

We have to make people sensitive to this issue as an issue like all the rest of them and talk about it whenever we get the chance, not just on a special day.

#### Pastoral experience influences faith leaders’ responses to HIV/AIDS

Many Pastors and Imams identified young age and lack of pastoral experience as impediments for faith leaders to address HIV/AIDS. Several pastors explained that the amount of time they spent leading a congregation impacted their comfort level discussing HIV/AIDS with their congregations. One young pastor expressed his reluctance to address HIV/AIDS with his congregants:

He [an older Pastor] can talk about AIDS because he’s been there, he’s established, folks have seen him for sixteen years, they can receive it from him. You and I, they may not be able to receive it [from us] now, so we need to teach it first in Bible Study. And then come back down the road and preach it.

An older pastor explained how age and pastoral experience emboldened him to address HIV/AIDS openly at church:

It has to do with how young you are and the age of the congregation’s members. I get away with some stuff now because of this gray hair and because of the 16 years I’ve been at my church. It’s the art form of pastoral theology, and it is leadership, and it is recognizing that you can’t USE your influence until you HAVE it, and it is learning how to utilize older people to help you and all of those things. I used to hate it [being a young pastor]. I was so glad when I turned forty!

### Recommendations for Enhancing HIV/AIDS Prevention in Faith Communities

#### Enhance leadership and HIV/AIDS advocacy efforts

There was strong consensus that the faith community should play a critical role in HIV/AIDS prevention in the African American community. Numerous participants explained:

Faith groups represent the most important and influential group that can reach black citizens. There will never be as many government offices in our community as churches!

The discussion “Look, HIV/AIDS is here. What do we do about it?” must begin with the most powerful voice in the community: the church. Let’s talk about it, let’s end the taboo. As long as we don’t talk about it and people die, it’s reducing our population and we’re not providing any kind of service to future generations.

I think it ought to match the role churches played in the Civil Rights Movement – with just that level of intentionality and intensity and consistency.

Participants explained that churches and pastors were among the most trusted leaders in the community:

The greatest benefit is that houses of worship are trusted. They’re safe places. So people, not only the congregation, but people from the community, people that haven’t even been to a church or a mosque or a temple, will see the need and they’ll trust the church before they trust the clinic and that’s the credibility that we bring.

If the church or the mosque or the synagogue were at the forefront of this issue, the community would say, “Wait a minute, if these individuals can wrap themselves around this issue, we need to become involved.”

#### Normalize HIV/AIDS testing and conversations about human sexuality

Almost all participants agreed that promoting more open dialogue about HIV/AIDS in the faith community would help reduce stigma.

If we keep discussing HIVAIDS– as clergy, as leaders in the community– we normalize the dialogue related to HIV.

We, who are Pastors, Imams, youth leaders, have to normalize the sex discussion. It should be normal that the Pastor in the pulpit talks about it, not using vernacular, but real terms so that it won’t feel foreign or apologetic. We don’t have the room now for a Pastor to stand in the pulpit, say “penis”, and say “I’m sorry.” It’s time for us to be able to talk about penises, vaginas, and breasts and anuses, and it be a regular part of how we talk, not vernacular, not funny, not apologetic, but just a regular part of what we say. And I think that helps other people embrace the language.

Most participants commented that pastors should help promote routine HIV testing, and that this could reduce AIDS-related stigma. Two pastor’s remarks reflected this common theme:

We need to standardize testing. One thing that we could do immediately is to encourage our congregations–everybody– get tested. To say, “We’re just going to do this as an exercise. Somebody next to you might have the virus and it will help them if you get tested. So, as a congregation, we’re just all going to get tested. We’re not dealing with risk factors. And we’re all going to get tested once a year.” That’s the one thing that we could do that doesn’t get into our doctrine about sexuality. It’s just about being responsible. And I’m going to start as the pastor by getting tested. The whole deacon and usher boards are going to get tested. Then you! That’s something concrete.

In my church, 99% of people love Reverend [Name]. If he started out saying, “I’m going to go get tested today,” you can believe me that 87% of that 99% are going to come get tested with him.

#### Overcome stigma with public health, human rights and healing approaches to HIV prevention

When asked how to overcome challenges with confronting AIDS stigma and addressing human sexuality in faith settings, a common theme that emerged was the importance of framing HIV/AIDS prevention as a public health and human rights issue. Participants frequently commented that couching HIV/AIDS in public health and human rights terms rather than exclusively in terms of human sexuality could help decrease stigma and increase interest in prevention.

I’ve found it helps to frame HIV/AIDS as a public health issue and human rights issue. Stigma and discrimination are human rights issues. It’s a public health issue because it is not stopping, so we must do something about it. In speaking with clergy, I find that if they can focus on that and not the sexuality, that this is a public health crisis in our communities, that we must do something about it. Then it becomes a human rights issue. Those are two buzz words that you really should think about. It really can make a difference in the way people hear it.

Several participants commented that HIV prevention campaigns frequently isolate the faith community by bundling discussions about HIV prevention with controversial discussions about sexual identity, and homosexuality in particular. One pastor explained this commonly cited challenge and suggested divorcing public health discussions about HIV/AIDS from these other issues:

We need to leave the margins out of this discussion, meaning any groups that are going to come and attach the gay and lesbian and civil rights issues to this testing day. We don’t want them there. I also don’t want my right-wing friends who want to come in here with anointing oil and heal everyone from homosexuality in here offending people! So let’s leave both of those groups out and let’s bring in groups that just want to do testing and talk about here are the ways you catch this disease, with dirty needles, unprotected sex, understand the statistically significantly proven dangers of anal sex as opposed to vaginal sex, understand the transmission of fluids and all of that, and only talk about THAT.

Similarly, one pastor explained that couching discussions about HIV/AIDS and scripture in terms of human healing and wellness provided an entry point for discussing HIV/AIDS with his congregation:

I always think about this the way Jesus dealt with leprosy. He put his hands on it. He touched people who had leprosy and it was no big deal. It was about how I’ll heal you from illness.

#### Promote diverse messages and approaches

There was a strong consensus among all participants that greater discussion and leadership was needed from faith leaders to effectively address the HIV/AIDS epidemic. However, there were wide-ranging suggestions for how faith-based institutions should enhance HIV prevention. Pastors generally agreed that diverse messages and tailoring HIV/AIDS programs to each faith institution were critical to fostering more widespread engagement. Several leaders felt they should lead HIV/AIDS discussions from the pulpit to help fight stigma:

We need to step up to the plate and start doing this from the pulpit, not from the back door having a little nice little ministry over here, but addressing these issues systemically….Any transformation in a church takes place through preaching. How about you think about doing a sermon series on health? Maybe the first Sunday you talk about how depression affects our work, families, etcetera. Then the third Sunday you’d talk about how everybody needs to take a sabbatical and then build themselves up. But also let the congregation know on the fourth Sunday, I’m going to deal with this issue of HIV AIDS. And tell them what the scripture is–maybe the text on leprosy–but my point is that you can deal with it through preaching.

If we don’t start preaching from the pulpit now on this issue, fifteen to twenty years from now we’re going to see more hospices, we’re going see more funeral parlors being built for the city of Philadelphia.

On the other hand, other participants acknowledged that discussing HIV/AIDS from the pulpit may not be a viable first step for many faith leaders. One participant remarked:

I had a pastor tell me that he could not deliver that from his pulpit. He would have to invite me in to do it in a workshop on a Saturday but he could not say it from the pulpit.

A few leaders noted that training faith leaders would better prepare them to engage their congregations in discussion about HIV and prevention campaigns. One pastor explained:

As clergy, we need to go to some type of mechanism where we can be taught about HIV and AIDS to debunk any misconceptions we have. Once we learn about it, then we can go out and teach others.

Many participants recommended integrating HIV/AIDS as topics for their health ministries to address. (Health ministries are committees within the church that minister and assist with congregants’ health issues):

It really needs to be part of the health ministry. You really can’t single out one health issue. You have to include all of them, diabetes, HIV AIDS. So it has to be part of the healthcare ministry and not necessarily just HIV AIDS. Because if it’s just HIV AIDS, then people say “I don’t have it.”

We do it for everything else. We have [prevention and education] programs for prostate cancer, we do it for diabetes, we do it for hypertension, all those things will kill you. So why not for HIV?

Participants suggested several other avenues for HIV/AIDS programs and preventions in the faith setting. Many leaders felt that discussion about HIV should also occur during non-Sunday church forums.

When a pastor is talking, it’s not for you to be throwing questions at him when he’s doing his sermon. But in an open conversation or sit down round table type of thing, this way people can ask you questions that might come to mind. And then they can get more information, more facts, the people who come to speak know more about it.

It’s important for us to have information sessions in intimate settings. For people to come in and for us to talk to them face to face in small groups, to hear it from their pastor, to hear somebody maybe from the congregation– somebody to talk to them and say “I have been infected or affected by HIV or AIDS.”

Other participants noted the need to move beyond the church to engage the entire community:

We have to stop thinking about church as being inside four walls. The church needs to move to the recreation centers, cafes, poetry slams, different music venues. Start getting involved in youth activities. Go to where they are, and as you entertain them, you’re educating them.

Many participants agreed that prevention programs should involve and target youth. Participants suggested that social media outlets such as Facebook could be utilized to engage youth.

We, our churches, and our community leaders need to take advantage of the technology that we have today that young people are involved with, such as Facebook and texting.

Most respondents mentioned that congregations could employ both active and passive prevention messaging. While most participants agreed that active programming can reach a larger proportion of congregants, many also suggested that other activities may help send important messages to congregants in more subtle ways. For example, one participant suggested that POZ magazine, a magazine that provides information to HIV-positive individuals, could be distributed or made prominently available in churches to help reduce stigma:

It might be useful for our churches to subscribe to POZ. And just have it available. Shared information, just out in the places where we keep stuff.

Another pastor explained that his church includes information in church bulletins to help normalize the discussion around HIV/AIDS:

We include vignettes where we get up and talk about something about HIV and AIDS, and we include some statistics or facts that are published in the bulletin.

Several participants underscored the importance of employing biblical scripture to start conversations about HIV/AIDS. Many pastors explained how they couched their HIV/AIDS conversations in scriptural contexts:

The Bible can be adapted to fit today’s society and that’s what we need to do, that’s what I’m trying to do, to fit with the vernacular and the lifestyle and the culture of today’s life.

It is natural to have sex. People are going to choose the partner they want to choose. Jesus hung out with prostitutes. And he never ever fussed at them for who they were. He fussed at religious people like us because we don’t want to do the ministering.

Leaders highlighted the importance of creating safe environments for people to disclose their HIV status, and for those leaders to start discussions for congregation-based HIV/AIDS programs:

The first thing is we have to create the environment. The second thing is to make it comfortable for those HIV-positive individuals in the congregation to testify that they have HIV. Because there are plenty of people in the congregation that have it. It only takes one to say, “I have it.” to allow others to feel comfortable to say that they have it.

Another participant noted how HIV-positive congregants can serve as role models:

We have an individual that has publicly identified himself as HIV-positive. He is a young person able to talk to our youth.

Many leaders felt strongly that abstinence still should play an important role in HIV prevention messages disseminated by the church. Many suggested encouraging abstinence while also promoting safe sex. One pastor explained:

The church really doesn’t give out condoms. People are going to have sex. Young people are going to do it no matter how much you say “Abstain, abstain.” And abstinence should be the message of the church. But are we being practical? It’s my duty as a preacher to tell people to abstain, but if they’re still having sex and they’re getting HIV, there has to be another way to handle this.

#### Promote interfaith collaboration

There was a common consensus that there was an important need for a collaborative, interfaith coalition to address the city’s AIDS epidemic, and a need to systematically engage the media in HIV prevention.

When you partner not only with the Baptist and not only with your faith or your denomination but ecumenically, we’re able to say,“Hey man, they’re doing something that’s working over there. And maybe we need to try to incorporate that or partner with the people at the mosque.” When you start talking ecumenically, people have a problem with that. “Oh, no we can’t do it. They don’t believe what we believe.” But it’s an epidemic that’s crossing the denominational lines. It doesn’t matter what your denomination is. This is affecting everybody. We have to work together!

It seems to me an association needs to emerge out of these focus groups–one to which we can all belong. It would be just great to say we are the association of HIV/AIDS for the city of Philadelphia.

One Imam suggested putting aside religious differences between Muslims and Christians to work collaboratively on addressing HIV/AIDS. He commented:

Brother, we’re humans first. And then we have responsibility to practicality and we’ve got to take this doctrine of theology and take it into the practical sense of doing the work. That’s all I’m concerned about. Like they say, “If your house is on fire, you don’t care who shows up as long as they’ve got the hose!” And guess what, brothers? With HIV/AIDS, THE HOUSE IS ON FIRE and we need to bring the hose!

## Discussion

Faith leaders understood how HIV is transmitted but were generally unaware of the gravity of Philadelphia’s epidemic and high HIV incidence in neighborhoods where churches are located. Geography has important impacts on HIV prevalence and has been associated with neighborhoods with higher rates of poverty in recent studies [57]. Lack of awareness about Philadelphia’s micro-epidemics underscores the importance of conducting more media and awareness campaigns about the local epidemic, with a focus on engaging community leaders in high incidence zones of the city to promote HIV testing and education. While faith leaders identified several important barriers to HIV prevention, they strongly believed that churches and mosques can and should play a critical role in addressing HIV/AIDS in the African American community, and in encouraging and normalizing HIV testing in particular.

The biggest challenges faith leaders identified for addressing HIV/AIDS in their communities were related to addressing human sexuality in faith-based contexts. Many participants commented that HIV/AIDS is still considered a “gay disease” in many African American social circles, explaining that fear of being perceived as gay and broader homophobia inhibited Pastors and Imams from discussing the HIV/AIDS epidemic more openly. This finding has been detailed in other research highlighting this challenge [23], [24], [58]. However, participants widely agreed that homosexuality was not the only sexuality-related challenge to addressing HIV/AIDS; discussing *human* sexuality more broadly was also commonly cited as a barrier. Many faith leaders agreed that addressing human sexuality in faith contexts was still often considered taboo and several often felt that discussing AIDS, human sexuality, risk behaviors, or condom use might be perceived as controversial in their churches or mosques. Faith leaders recommended that we not let discussions related to behaviors inhibit the response of faith institutions’ to the epidemic; some suggested embracing discussions related to human sexuality, while others suggested focusing greater efforts on less divisive prevention messages, like social justice, human rights, and HIV testing.

Scarce resources were another important barrier to addressing the epidemic. Several pastors candidly explained their fears that discussing HIV/AIDS at church might have detrimental impacts on church attendance and tithing. This highlights the fact that economic considerations impact faith leaders’ decisions about addressing the epidemic: discussing AIDS has serious economic implications for some faith leaders. Many participants also commented that creating programs to address HIV/AIDS required staff and financial resources that their institutions did not have. Understanding these economic realities has important implications for planning HIV prevention programs for faith institutions. Many pastors recommended allocating greater public resources for HIV prevention to faith institutions, particularly related to HIV testing and education.

Several younger pastors commented that they did not feel emboldened to discuss AIDS at church, fearing they might alienate parishioners who preferred not to discuss issues related to human sexuality. There was a common consensus that discussing controversial topics such as HIV/AIDS was easier for more established faith leaders with larger followings and more experience. This suggests that engaging more experienced pastors to help jumpstart public dialogue about HIV/AIDS might help normalize discussions about HIV/AIDS with the faith community.

Much research about engaging the African American faith community in HIV prevention focuses on homophobia, barriers to engaging faith leaders in the fight against HIV/AIDS, and the faith community’s muted response to the AIDS epidemic [21]–[24], [26], [59]–[61]. This article adds to a growing body of literature which highlights important opportunities for engaging the African American faith community in HIV prevention [25], [44] and builds on that literature by highlighting faith leaders’ normative suggestions for how to engage the faith community in HIV prevention.

Faith leaders offered important normative suggestions for how to address the HIV/AIDS epidemic in faith settings. First, nearly all participants recommended that Pastors and Imams play a critical leadership role in addressing the epidemic and should break the silence about HIV/AIDS to help normalize the community discussion about HIV/AIDS.

Leaders offered diverse opinions about how to jumpstart the faith community’s response to the epidemic. Some favored a more active approach, and HIV testing in particular was strongly endorsed by most leaders. Many believed that the Pastor or Imam should start the dialogue by preaching about HIV/AIDS, testing publicly, and subsequently encouraging the entire congregation to get tested. Many participants recommended citing CDC guidelines calling for routine HIV testing was the best way to frame discussions about HIV/AIDS, rather than couching conversations in the context of sexual orientation, sexual risk behaviors, or human sexuality.

HIV testing is a highly effective HIV prevention strategy, as individuals who test positive tend to reduce HIV risk-taking behaviors [62]. Moreover, HIV testing is the first step in linking HIV positive individuals to treatment and care services; adhering to highly active anti-retroviral therapy (HAART) dramatically reduces the chances that HIV positive individuals will transmit the HIV virus to others [63]. HIV screening is also highly cost effective [64]. Faith leaders noted that focusing on expanding and normalizing HIV testing as the cornerstone for expanding HIV prevention programs with faith institutions may also avoid many of the barriers associated with behavioral interventions focused on reducing sexual risk behaviors that have historically inhibited the faith-based response to the AIDS epidemic. Focusing on normalizing and expanding HIV testing and retention in AIDS care would also be a strong compliment to the CDC’s High-Impact Prevention Strategy [65], as well as President Obama’s National AIDS Strategy goal of reducing racial disparities in HIV infection [30].

Pastors had diverse opinions about discussing condom use: some felt comfortable discussing condoms, others mentioned they would like to be able to discuss condoms with their congregations but did not feel comfortable, and others endorsed abstinence-only prevention messages. However, many participants recommended that faith leaders could play a key role in helping normalize discussions about human sexuality in their houses of worship. This underscores the importance of adopting a variety of HIV prevention messages that will resonate with faith leaders, which should potentially include, but should not be limited to, discussions about abstinence.

Many leaders suggested that scripture was a useful vehicle for drawing historical parallels between Jesus’ service to poor, marginalized and stigmatized populations and the need for religious institutions to embrace those affected by HIV/AIDS. Other participants believed that discussions should start either with congregant testimonials, within Health Ministries that can integrate HIV/AIDS into their curricula and programs, or through community outreach programs. Still others believed that subtle affirming messages in church bulletins or handouts could help send the signal that institutions embrace those living with or affected by HIV/AIDS. Many leaders believed some or all of these programmatic approaches were critical for engaging their congregations in the fight against AIDS.

Taken together, these findings suggest that “diffusion of evidence-based interventions” (DEBIs) (http://www.effectiveinterventions.org/) disseminated by the CDC for HIV prevention may be impractical for faith-based settings. Instead, a more diverse approach that includes tailoring programs to each faith institution will be critical for building public health alliances and HIV/AIDS programs in partnership with African American faith institutions. This may include programs that promote abstinence and delayed sexual debut.

An important suggestion that emerged was framing HIV/AIDS as a social justice, human rights and public health issue rather than an issue about sexual orientation. Leaders recommended that couching HIV prevention discussions in these terms or in the context of human and spiritual healing might help mitigate some of the challenges associated with discussing human sexuality in religious contexts. Leaders also commonly believed that many HIV/AIDS discussions have historically been tied to sin, homosexuality and curing homosexuality; several remarked that de-linking these issues from the AIDS discussion and focusing on HIV testing in particular might help mitigate the challenges and controversies that have historically limited the faith-based response to HIV/AIDS in the African American community. This big-tent, public health approach that focuses on HIV testing and leadership rather than HIV risk behaviors presents an important framework for engaging the faith community for policymakers, researchers and practitioners alike.

This qualitative study has several limitations. First, approximately half of our sample was based on churches and mosques that had existing relationships with the Mayor’s Office of Faith Based Initiatives or the principal investigator and are not necessarily representative of the broader Philadelphia faith community or the national African American faith community. Just under half of participants were affiliated with the two largest African American Christian denominations in Philadelphia: Baptists and African Methodist Episcopal churches. We do, however, note that leaders of most of Philadelphia’s largest African American religious congregations did participate in the study and there was diversity among the Christian denominations represented. Their perceived barriers and recommendations for faith leaders to engage in HIV prevention may not be nationally representative of all African American faith leaders’ opinions.

This study nevertheless highlights a number of important barriers and recommendations for engaging African American faith leaders in HIV prevention. Although the faith community has played an overwhelmingly important role in social justice and African American community life, few interventions have been identified that successfully engage African American faith institutions in HIV/AIDS prevention. Our findings challenge the commonly held belief that African American faith institutions are unwilling or reluctant to address the HIV/AIDS epidemic [66]–[68]. In contrast with other research that highlights problems associated with engaging African American faith institutions in the fight against HIV/AIDS, our findings suggest that influential pastors from large urban centers heavily impacted by the AIDS epidemic are willing to address the HIV/AIDS epidemic in their own houses of worship, are willing to promote and host HIV testing events, and are also willing to work collaboratively in interfaith coalitions to address the epidemic.

Other research finds that culturally tailored HIV prevention interventions should engage community leaders and members, build upon community strengths, and capitalize on cultural pride [32], [36], [69]. Using community leaders for HIV prevention can also help mitigate the heavy stigma often associated with HIV/AIDS [70]–[72]. Understanding faith leaders’ perceived barriers and proactive recommendations for effectively engaging faith institutions in HIV prevention are critical first steps for developing structural and community-based HIV prevention interventions intended to address racial disparities in HIV infection. The positive, proactive, and concrete recommendations presented in this study provide a roadmap that can inform public policy about how to effectively collaborate with African American faith institutions to reduce the United States’ racial disparities in HIV infection.
